# Rationally designed sodium thiosulfate-loaded solid lipid nanoparticles for inner ear delivery and prevention of medication-induced ototoxicity

**DOI:** 10.1039/d5tb01324k

**Published:** 2025-10-27

**Authors:** Brototi Chakrabarty, Neeraj S. Thakur, Aditya D. Joshi, Vibhuti Agrahari

**Affiliations:** a Department of Pharmaceutical Sciences, The University of Oklahoma 1110 North Stonewall Avenue Oklahoma City OK 73117 USA Vibhuti-Agrahari@ou.edu; b Department of Veterinary Physiology and Pharmacology, Texas A&M University College Station TX 77843 USA

## Abstract

Medication-induced ototoxicity (MIO) results from treatment regimens such as aminoglycosides and platinum-based drugs, leading to sensory hair cell damage in the inner ear, which is responsible for converting mechanical sound vibrations to the electrical signals for hearing. Our study aims to develop biomaterial-based localized drug delivery systems of therapeutics for the protection of cochlear hair cells. In this study, we developed sodium-thiosulfate (STS)-loaded solid–lipid-nanoparticles (SLNs) and tested them against cisplatin (CisPt)-induced ototoxicity. STS-SLNs were synthesized by the double emulsion evaporation technique followed by characterization using dynamic light scattering (DLS), nanoparticle tracking analysis (NTA), and transmission electron microscopy (TEM). The optimized nanoparticles exhibited optimal physicochemical properties, and stability, including particle size (∼92.3 ± 0.8 nm), polydispersity index (<0.3), zeta potential (−13.23 ± 2.07 mV), and encapsulation efficiency (45.48 ± 5.87). TEM analysis confirmed the STS-SLNs spherical morphology. The STS-SLNs showed sustained release of STS from SLNs with an *n* value of 0.09 (Fickian diffusion) determined using the Korsmeyer–Peppas model. Cellular uptake studies with House Ear Institute-Organ of Corti (HEI-OC1) cells using Coumarin-6-tagged STS-SLNs showed a maximum uptake at 1 hour *via* clathrin-mediated endocytosis. The STS-SLNs displayed antioxidant potential in reactive oxygen species (ROS) scavenging assays, and enhanced cell viability in live/dead assays compared to CisPt treatment alone. The molecular signaling pathways were investigated by assessing the expression of STAT3 and Nrf2 pathways in HEI-OC1 cells. STS-SLNs significantly reduced STAT3 and P-STAT3 expression compared to the CisPt-treated group, suggesting a protective effect against CisPt-induced oxidative stress *via* the STAT3 pathway. STS-SLNs effectively mitigated medication-induced (CisPt) cell damage in auditory cells, highlighting their therapeutic potential for local delivery to the inner ear.

## Abstract 2

Our study culminates in the comprehensive development and evaluation of the sodium thiosulfate-loaded solid lipid nanoparticle (STS-SLN) delivery system for mitigating medication-induced hearing loss, employing cisplatin as a model drug.

## Graphical abstract

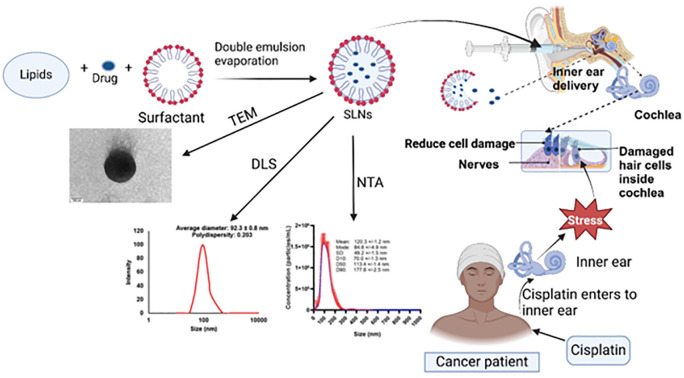

## 1. Introduction

One of the most common sensory impairments in the world today is hearing loss. Based on the most recent WHO statistical data, rehabilitation is necessary for 430 million people worldwide, or more than 5% of the population (432 million adults and 34 million children), who suffer from deafness. Ototoxic medications, including aminoglycoside antibiotics, cisplatin (CisPt), and loop diuretics, contribute significantly to hearing loss. Studies estimate that 20–60% of patients receiving chemotherapy experience some degree of ototoxicity, with children being particularly vulnerable. Additionally, about 2–4% of adults on long-term aminoglycoside therapy develop irreversible hearing impairment. The global burden of ototoxicity is expected to rise with the increased use of these medications in cancer treatment and infection management.^1–4^ Around 700 million people, or one in ten, are predicted to have debilitating hearing loss by the year 2050.

For our study, we selected CisPt as a model drug, which is commonly used to treat cancers of the ovary, testicles, cervical region, non-small cell lung, bladder, and head and neck, and acts as an ototoxic agent. Side effects like nephrotoxicity, neurotoxicity, and ototoxicity can occasionally limit the CisPt treatment. The estimated ototoxicity burden caused by CisPt affects 36% of adult cancer patients and 40–60% of pediatric patients.^5^

The death of sensory hair cells of the Organ of Corti (located within the cochlea), primarily outer hair cells (OHC) but also inner hair cells (IHC) *via* reactive oxygen species (ROS) induced apoptosis and necrosis, is the major cause of CisPt-induced hearing loss. The structures of mitochondrial DNA (mDNA) and nuclear DNA (nDNA) are altered by CisPt-induced adduct formation. The caspase and MAPK/JNK-dependent pathways are triggered by oxidative stress and the release of inflammatory mediators in reaction to the damage caused by CisPt-induced cell death of hair cells.^6^ CisPt can enter hair cells through passive diffusion and is responsible for triggering cell death *via* oxidative stress and mitochondrial dysfunction. Moreover, CisPt may potentially get access to cochlear target cells *via* transporters. It has been proved that CisPt-induced hearing loss is associated with ferroptosis, necroptosis, and increased levels of ROS as well as nitric oxide (NO).^1,7^ There is no mechanism for removing CisPt from the inner ear after it enters the cochlea.^7^

Systemic drug delivery presents several challenges when it comes to protecting the cochlea from CisPt-induced damage. The blood-labyrinth barrier prevents drugs from entering the cochlea when administered systemically.^8^ Secondly, the arteries into the inner ear are composed of incredibly thin terminals that are responsible for slow blood flow and inadequate collateral circulation. Among the pharmacological difficulties, the drug's poor permeability, which restricts its passage through the round window membrane (RWM) into the inner ear, instability, and incapacity to efficiently assemble in both internal and external lymphatic fluids, as well as its nontargeted delivery, are the four most common factors affecting the drug absorption.^9^ However, these obstacles may be overcome by administering otoprotective drugs locally. Compared to systemic administration, there is a higher concentration of medication at the target site *via* local administration while avoiding systemic side effects. Nanotechnology enables the effective delivery of protective drugs into the cochlea through the RWM.^10^

Sodium thiosulfate (STS) is a hydrophilic drug molecule that can prevent CisPt-induced hearing loss, as reported through animal and human studies. The administration of STS after CisPt has been proven to significantly reduce the risk of hearing loss in children who were receiving CisPt.^11,12^ Brock *et al.*, in 2018 again confirmed these findings in a trial with children who had standard-risk hepatoblastoma, showing that the addition of STS (intravenously) after CisPt chemotherapy reduced the prevalence of deafness without affecting life expectancy.^13^ However, the systemic administration of STS may reduce the antitumor activity of CisPt, and this drawback can be minimized by local administration (intra-tympanic) to the inner ear.^14^ Wang *et al.*, demonstrated that local administration of STS in the guinea pig cochlea prevented hearing loss without affecting the antitumor effect of CisPt.^15^

The special ability of solid lipids to enhance the bioavailability of hydrophilic drugs makes them a suitable vehicle for preparing nanoparticles of these drugs. With solid lipid nanoparticles (SLNs), drug release is more stable and prolonged than conventional colloidal carriers because they reduce problems like drug leakage and burst release. Various techniques, such as double emulsification and microemulsion, have been developed to enhance drug entrapment efficiency despite the inherent challenge of incorporating hydrophilic molecules into the lipid matrix of SLNs. Through the stable incorporation of hydrophilic drugs made possible by these methods, the delivery of water-soluble therapeutic agents can be enhanced. Thus, SLNs are promising vehicles for achieving local and controlled inner ear drug delivery. For example, doxorubicin has been successfully encapsulated using a w/o/w emulsion, achieving significant entrapment efficiencies.^16^ Our study aimed to develop an STS-loaded SLN (STS-SLN) nanodrug to treat medication-induced ototoxicity (MIO). We have prepared and tested a drug delivery system targeting the inner ear with the combination of stearic acid and lauric acid as solid lipids.

## 2. Materials and methods

### 2.1. Materials

Sodium thiosulfate (STS, Thermo Fischer), *cis*-diammineplatinum(ii) dichloride (CisPt, TCI), chlorpromazine (CPZ, TCI), methyl-β-cyclo-dextrin (MβCD, TCI), amiloride (AML, TCI), genistein (GNT, TCI), triethylamine (TEA, Fischer Chemical), methanol (Fischer Chemical), dichloromethane (DCM, Fischer Chemical), acetonitrile (ACN, Fischer Chemical), coumarin-6 (Thermo Fischer), Tween 60 (Thermo Fischer), stearic acid (Thermo Fischer), lauric acid (Thermo Fischer), thiazolyl blue tetrazolium bromide (MTT, Thermo Fischer), and anhydrous DCM were purchased from Millipore Sigma, USA. The cell culture media, reagents, and phosphate buffer saline (PBS) were procured from Millipore Sigma, USA.

### 2.2. Synthesis and formulation optimization

To identify the most suitable nanocarrier for sodium thiosulfate (STS), we first carried out preliminary trials with PCL-PEG, PLA-based diblock and triblock polymeric nanoparticles. These experiments were designed to assess particle size, encapsulation efficiency, and reproducibility across different polymer matrices. Based on these preliminary investigations, we subsequently developed solid–lipid-nanoparticles (SLNs), which offered a more promising platform for STS delivery and were further optimized and investigated in detail.

#### Optimization of STS-SLNs

The formulation process was systematically optimized by varying key parameters using a core formulation composed of water, an emulsifier, two solid lipids, and sodium thiosulfate (STS) as the active drug.

##### Lipid screening

Lipid screening was done to determine the optimal miscibility of the active ingredient with different lipid combinations. Briefly, three distinct formulation compositions were evaluated, each containing 2 mg of STS and 1% polyvinyl alcohol (PVA) as a base. Formulation-1 combined 10 mg each of stearic acid and palmitic acid; formulation-2 used 10 mg each of stearic acid and lauric acid; and formulation-3 contained 10 mg each of stearic acid and hexadecyl palmitate (Table 1).

**Table 1:** Formulation optimization: lipid name and composition

Formulation-1		Formulation-2		Formulation-3	
Lipid name	Amount	Lipid name	Amount	Lipid name	Amount
STS	2 mg	STS	2 mg	STS	2 mg
Stearic acid	10 mg	Stearic acid	10 mg	Stearic acid	10 mg
Palmitic acid	10 mg	Lauric acid	10 mg	Hexadecyl palmitate	10 mg
PVA	1%	PVA	1%	PVA	1%

##### Surfactant selection

Formulation-2 worked well to prepare the solid–lipid-nanoparticles without any precipitation. After selecting the lipid composition, we selected the surfactant and its concentration to achieve a stable nanoformulation (Table 2), keeping the lipid composition and amount the same as in formulation 2. For each surfactant variation, we analyzed the nanoparticle size and polydispersity index using dynamic light scattering (DLS), as these parameters directly influence the suspension stability of the SLNs.

**Table 2:** Lists of surfactants with concentration

Name of surfactant	Concentration
PVA	1% and 2%
P-188	1%
Tween-20	1%, 5% and 10%
Tween-80	1%, 5% and 10%
Tween-60	0.5%, 1%, 5% and 10%

##### Nanoparticle synthesis procedure

SLNs were synthesized using the double emulsion solvent evaporation method following previous procedures with modification.^16^ The optimized formulation (Table S1) includes lipids (stearic acid and lauric acid; 10 mg each), which were dissolved in 1 mL of DCM to prepare the organic phase (O). The aqueous phase (W1) was prepared by dissolving STS (2 mg) in 100 μL deionized (DI) water. The second aqueous phase (W2) was prepared using the 5% solution of Tween 60 in DI water. The STS solution (W1) was vigorously mixed with lipids using ultrasonication (FB120, Fischer Scientific USA) for 30 s at 65% amplification to obtain a water-in-oil (W1/O) emulsion. The primary W1/O emulsion was then vigorously mixed with W2 (5% surfactant, 4 mL) using ultrasonication for 120 s (pulse on 60 s, off 5 s) to prepare the final W1/O/W2 emulsion. The final emulsion was placed under stirring for 3 hours to let the DCM evaporate. STS-SLN colloidal solution was then filtered through centrifugation at 4000*g* for 1.5 h (SORVALL LYNX 4000, Thermo Scientific). The pellet was resuspended in deionized water (500 μL) and stored at 4 °C before further characterization.

### 2.3. HPLC methodology

A reverse-phase HPLC technique that was modified from earlier research was used to evaluate STS detection.^17^ In brief, the analysis was performed using an Agilent 1260 Infinity HPLC system equipped with a variable wavelength detector (VWD). The chromatographic separation employed a Phenomenex Columbus C-18 column (5 μm, 100 × 4.6 mm) as the stationary phase. The mobile phase consisted of 100% methanol (MP-A) and 0.1% TEA in water adjusted to pH 7.4 (MP-B), running in an isocratic mode at a ratio of 15 : 85 (MP-A : MP-B) with a flow rate of 0.5 mL min^−1^. All analyses were conducted at room temperature with UV detection at 210 nm over a 4-minute run time. For quantitative analysis, a standard calibration curve was established using STS solutions prepared in MP-B at concentrations ranging from 0.1953125 to 100 μg mL^−1^. The resulting calibration curve demonstrated excellent linearity with an equation of *y* = 27.354*x* + 3.2019 and a correlation coefficient (*R*^2^) of 1.00. Method validation was performed according to ICH guidelines, analyzing three samples of each concentration daily over three consecutive days to ensure both qualitative and quantitative reliability. This comprehensive validation protocol confirmed the suitability of the HPLC method for accurate STS detection and quantification in subsequent experiments.

### 2.4. SLN characterization: particle size, polydispersity, and zeta potential

The comprehensive characterization of STS-loaded solid lipid nanoparticles (STS-SLNs) was conducted using multiple complementary analytical techniques. Particle size and polydispersity were evaluated using DLS with a Zetapals instrument (Brookhaven Instruments, Holtsville, NY). Surface charge properties were assessed through zeta potential measurements using the same instrument. Nanoparticle tracking analysis (NTA) was performed using a NanoSight-NS300 (Malvern Inc., UK) to confirm particle concentration and size distribution data. Morphological examination was conducted using transmission electron microscopy (TEM; JEOL 201F, Japan).

For DLS analysis, a 1 : 20 dilution ratio was employed by adding 50 μL of nanoparticle suspension to 950 μL of deionized water. This same dilution served as a stock solution for subsequent analyses. NTA required further dilution, where 2 μL of the stock solution was diluted to 1 mL with deionized water. For zeta potential measurements, 60 μL of nanoparticle stock suspension was diluted with 1.4 mL of deionized water. For TEM analysis, 5 μL of nanoparticle suspension was applied to a TEM grid and allowed to settle for one minute. The excess liquid was then removed, and the grid was washed twice with deionized water. Staining was performed by applying phosphotungstic acid (PTA) for one minute, followed by a single deionized water wash. After air-drying for 5 minutes, the samples were examined by TEM to visualize the morphology of the nanoparticles.

### 2.5. Encapsulation efficiency and drug loading study

The concentration of encapsulated STS into respective SLN was determined using a standard indirect method. Briefly, the concentration of STS in the supernatant solution was determined using HPLC (quantification was performed according to HPLC methodology, Section 2.3). Each measurement was performed in triplicate. The percentage of encapsulated efficiency (EE%) and percent drug loading (DL%) were calculated using the following formulas:1
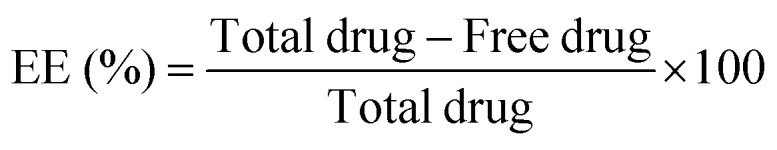
2
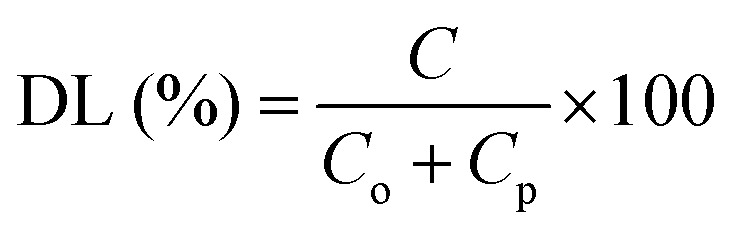
where *C* is the concentration of the drug in the NPs, *C*_o_ is the concentration of added STS and *C*_p_ is the concentration of added polymer.

### 2.6. *In vitro* release study and release kinetics

The release study of the STS-SLNs was evaluated using a dialysis membrane, while PBS (pH 7.4, Sigma, USA) was used as the receiving medium. Following preparation, the STS-SLN suspension was adjusted to a final volume of 1 mL and transferred into a 3.5 kDa molecular weight cutoff dialysis membrane (Spectra/Por® 3). The membrane was sealed by clipping both ends and then immersed in 50 mL of PBS. Release kinetics were monitored by withdrawing 1000 μL samples from the receiving medium at predetermined time intervals (30 min, 1, 2, 4, 6, 8, 24, 48, 72, and 96 h). After each sampling, an equal volume of fresh PBS was added to maintain sink conditions. The STS concentration in each collected sample was quantified using the previously validated HPLC method. The release data were analyzed using the Korsmeyer–Peppas mathematical model (eqn (3)) to determine the release mechanism and kinetics of STS from the STS-SLNs.^18^

3 *F* = (*M*_*t*_/*M*_∞_) = *K*_1_*t*^*n*^

where *M*_*t*_ is the mass of the drug released at time *t*; *M*_∞_ is the mass of the total drug; *k*_1_ is the release rate constant; *n* is the release mechanism exponent.

### 2.7. Storage stability and freeze-drying studies

The storage stability and freeze-drying study were performed following a previously published method from our lab with very few modifications.^19^ The stability of STS-SLNs was studied at 4 °C for two months in DI water. 300 μL of STS-SLNs was mixed with 300 μL of DI water and stored at 4 °C and the size and polydispersity were determined on the 0, 7, 14, 30, and 60th days.

For lyophilization studies, freshly prepared STS-SLNs were frozen at −80 °C overnight and subjected to lyophilization in the presence of different cryoprotectants (mannitol, sucrose, and trehalose). Different amounts (1, 2.5, 5, 7.5, and 10% w/v) of cryoprotectants were added to the solution of STS-SLNs and lyophilized using a Freeze Dryer BK-FD10P, JIAN MEDICALCO., LTD. After lyophilization, samples were reconstituted in deionized water, and particle size and polydispersity index (PDI) were measured by DLS to assess formulation stability.

### 2.8. *In vitro* assessments using HEI-OC1

#### 2.8.1. Cellular toxicity of STS and STS-SLNs towards HEI-OC1 cells

A conventional MTT test was used to measure cellular metabolic activity as a sign of cell viability, proliferation, and cytotoxicity after cells were exposed to SLNs to examine the cytotoxicity of SLNs. The HEI-OC1 cells were seeded in 96-well plates at a density of 1 × 10^4^ cells per well and kept in an incubator for around 65 h at 33 °C (5% CO_2_). The cells in every column were then incubated in medium (100 μL) with various treatment components (column 1: blank medium, column 2: blank medium only, column 3: STS-NPs equivalent to 50 μM of STS per well). After 5 h of incubation, columns 2 and 3 received 100 μL of medium with 30 μM CisPt. The cells were incubated at 33 °C in 5% CO_2_ for 36 hours. Subsequently, 100 μL MTT containing medium (10 μL, 5 mg mL^−1^ MTT reagent in 90 μL growth medium; MTT assay kit, Roche, Germany) was added to each well and incubated for another 5 h. The solubilizing buffer (100 μL) in each well was added and the plate was incubated overnight. After incubation, the absorbance was recorded at 570 nm using a multi-well plate reader (Synergy 2, BioTek, USA).

#### 2.8.2. Cellular internalization study

1 mg of Coumarin-6 (C-6) was added to the colloidal solution of blank SLNs, and the resulting mixture was agitated overnight (150 rpm) at 4 °C in a sealed vial. To eliminate the unloaded C-6, the resultant colloidal mixture with C-6 loaded SLN NPs (C-6-NPs) was centrifuged for 5 minutes at 2000 rpm. The C-6-NP-containing supernatant was utilized as a stock solution in the experiment. In a culture flask, the HEI-OC1 cells were grown for 7 days at 33 °C in complete growth media. After that, the cells were washed with new media and gathered in a centrifuge tube. The cells were seeded in 96-well plates (1 × 10^4^ cells per well in 100 μL media) and kept in an incubator for 2 days at 33 °C. After 2 days, the culture medium of each well was replaced with the media containing C-6-NPs (1 : 100; 7 μL C-6-NPs stock diluted up to 7 mL using culture media). The plates were kept at 33 °C, 5% CO_2_ for incubation. After 15 min, the media from the wells of the 1st column were removed. The cells were washed twice with fresh DPBS, and 100 μL fresh DPBS was added to each well of the 1st column and kept in an incubator. The same procedure was followed for six more consecutive columns (2–7) after 0.5, 1, 2, 3, 4, and 5 h of incubation, respectively. After that, the fluorescence intensity was recorded at *λ*_ex/em_ 530/615 nm using a multi-well plate fluorescence spectrophotometer (Synergy 2, BioTek, USA).

The cells were grown as discussed above to confirm the cellular uptake pathway and treated with different uptake inhibitors (CPZ, MβCD, AML, and GNT) at a 50 μM mL^−1^ concentration for 2 h. Then, 100 μl C-6-NPs (1 : 100; 5 μL C-6-NPs stock diluted up to 5 mL using culture media) was added to each well and incubated for 4 h. The cells were then washed with DPBS, and the fluorescence intensity was recorded using a multi-well plate reader as discussed above.

#### 2.8.3. Intracellular ROS scavenging capability study

The ROS scavenging ability of the STS-SLNs was determined through the DCFH_2_-DA assay.^20^ The HEI-OC1 cells were seeded in 96 well plates at a density of 1 × 10^4^ cells per well and kept in an incubator for around 65 hours at 33 °C (5% CO_2_). Then, the cells were treated with a different drug regimen (column 1: blank medium, column 2: blank medium only, column 3: STS-NPs equivalent to 50 μM of STS per well) and incubated again for 5 h. After 5 h, in all columns (except for column 1), 100 μL of medium containing 30 μM CisPt was added and incubated for the next 24 h. Then, the medium was removed, and 100 μl medium containing DCFH_2_-DA (10 μM) was added to each well and incubated for 30 minutes. Next, the cells were washed with PBS (2 times) and fresh PBS was added. The fluorescence intensity was measured using a multi-well plate fluorescence spectrophotometer (Synergy 2, BioTek, USA; *λ*_ex_/*λ*_em_ for DCF were 480/530 nm).

#### 2.8.4. Live–dead cell assay

To analyze live–dead cells, the cells were seeded in 96 well plates at a density of 1 × 10^4^ cells per well and kept in an incubator for around 65 hours at 33 °C (5% CO_2_). Then, the cells were treated with a different drug regimen (column 1: blank medium, column 2: blank medium only, column 3: STS-NPs equivalent to 50 μM of STS per well) and incubated again for 5 h. After 5 h, in all columns (except for column 1), 100 μL of medium containing 30 μM CisPt was added and incubated for the next 24 h. Then, the medium was removed, and 100 μl medium containing propidium iodide (PI) (1 μg mL^−1^ media) was added to each well and incubated for 30 minutes. Next, the cells were washed with PBS (2 times) and fresh PBS was added. Then, the fluorescence intensity was measured using a multi-well plate fluorescence spectrophotometer (Synergy 2, BioTek, USA; *λ*_ex_/*λ*_em_ for PI were 530/615 nm).

#### 2.8.5. Western blot analysis

HEI-OC1 cells were seeded into a 6 well plate (1 × 10^5^ per well) and incubated overnight at 33 °C (5% CO_2_). The treatment groups were an untreated control, CisPt only, and STS-SLNs. CisPt was added after 4 h incubation of STS treatment. After 2 days of treatment, the medium was removed from the wells and cells were washed (2×) with PBS. After washing, a 100 μL mixture of 2× blue (90 μL, for recipe, see the SI, Section S4) and β-mercaptoethanol (10 μL) was added. Sterile cell scrapers were used to collect the cells in a microcentrifuge tube. Then the samples were heated for 5 min at 100 °C. The samples were either kept at −20 °C for later use or loaded onto the gel (SDS-PAGE). The samples were run in a precast SDS-PAGE gel (4–15% Mini-PROTEAN® TGX™ Precast Protein Gels, BIO-RAD). Using the Trans-Blot® Turbo™ Transfer System (BIO-RAD), the samples were then transferred to the PVDF membrane after running. Bovine serum albumin (BSA) (5%) in TBS-T with 0.1% Tween 20 was used to block the membranes for one hour at room temperature. After washing, the membranes were incubated for the entire night at 4 °C with the appropriate primary antibodies, named signal transducer and activator of transcription 3 (STAT3) (12640s, 1 : 1000, Cell Signaling Technologies), nuclear factor erythroid 2-related factor 2 (Nrf2) (#12721s, 1 : 1000, Cell Signaling Technologies), and phosphorylated form of STAT3 (P-STAT3) (#9145s, 1 : 1000, Cell Signaling Technologies), which was diluted in BSA (5%) in TBS-T. The membrane was then washed with TBS-T (4 times every 5 min) and incubated again with goat anti-rabbit (#12,004,161, BIO-RAD, 1 : 2500) secondary antibodies for 1 h. Then, the membranes were washed using TBS-T (4 times every 5 min), and Biorad's ChemiDoc Imaging System was used to image the bands that correspond to the target expression. The membrane was washed using TBS-T (2 times every 5 min) and treated with control β-actin (3800739, MAB1501, Millipore, 1 : 1000) and the same washing and incubation procedure was followed as for the primary antibody. Secondary goat anti-mouse antibody (926-32210, Li-COR, 1 : 2500) was added and the Biorad's ChemiDoc Imaging System was used to image the bands. Image lab software of BoRAd was used to analyse the data.

### 2.9. Statistical analysis

The statistical analysis of data was carried out using ANOVA (one-way) with GraphPad Prism 10 (San Diego, USA). The tests were validated using Šidák's multiple comparison *post hoc* test where *p* < 0.05 was considered significantly different. The graphs and figures were drawn using GraphPad Prizm 10, excel.

## 3. Results and discussion

### 3.1. Synthesis and formulation optimizations of the STS-SLNs

#### 3.1.1. Preliminary evaluation of polymeric nanocarriers

In the initial stage of formulation development, STS was encapsulated into different polymeric nanocarriers, including PCL–mPEG, PCL–PEG–PCL, and PLA–PCL–PEG, using the double-emulsion (w/o/w) solvent evaporation method. Although nanoparticles were successfully obtained, all three polymer systems demonstrated significant limitations. The mean particle size consistently exceeded 300 nm, the encapsulation efficiency remained low (approximately 20–30%), and drug loading was below 3%. In addition, poor batch-to-batch reproducibility was observed.

These findings highlighted the difficulty of efficiently encapsulating highly water-soluble drugs like STS within polymeric matrices. They indicated that polymeric nanoparticles may not be the most suitable method for STS. The limited performance of polymeric carriers can be caused by the hydrophilic nature of STS, which mainly remains in the external aqueous phase during double-emulsion, leading to lower encapsulation efficiency and drug loading. In addition, the semicrystalline structure of PCL and PLA restricts the entrapment of small hydrophilic molecules, resulting in drug leakage and instability, as similarly reported for other water-soluble drugs.^21^ This results in poor reproducibility and aggregation observed in our polymeric formulation, directing us to develop SLNs as a more suitable platform.

#### 3.1.2. Optimization of the synthesis of the STS-SLNs

**Fig. 1:**
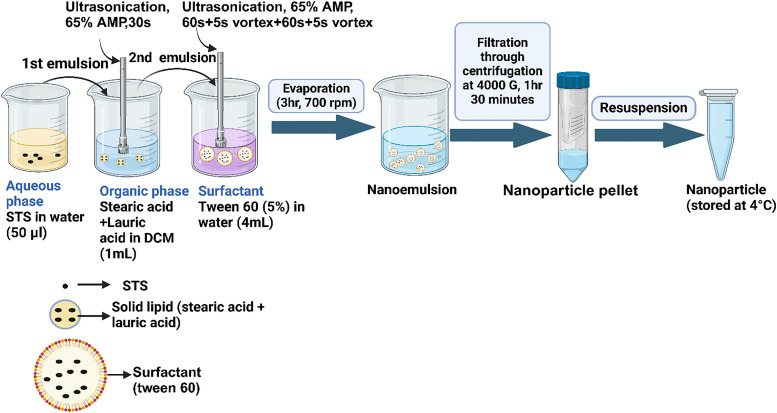
Schematic representation of the optimized synthesis procedure of the STS-SLNs.

The selection of an appropriate synthesis method is crucial for developing SLNs with optimal pharmaceutical properties. Various techniques, including emulsification-solvent diffusion, microemulsion, hot homogenization, high-pressure homogenization, and double emulsion solvent evaporation, offer different advantages for nanoparticle formulation.^22^ To synthesize STS-SLNs (Fig. 1), we employed the double emulsion solvent evaporation technique due to its particular suitability for encapsulating hydrophilic drugs like STS.^23^ This method creates a water-in-oil-in-water (w/o/w) double emulsion where the hydrophilic drug is first dissolved in an internal aqueous phase and emulsified into a lipid phase containing organic solvent, forming a primary water-in-oil emulsion. This primary emulsion is then further emulsified into an external aqueous phase containing stabilizers to prevent drug leakage. During solvent evaporation, the lipid aggregates solidify to form stable nanoparticles with the hydrophilic drug entrapped within the solid lipid matrix, protecting against degradation and enabling controlled release.^24^

For lipid selection, we evaluated three different lipid compositions to identify the optimal formulation for STS-SLNs (Table 1). After centrifugation at 18 000 rpm for 45 minutes, only formulation-2, containing a combination of stearic acid and lauric acid, successfully produced nanoparticle pellets, while formulations-1 and 3 failed to yield stable nanoparticles (Table 3)

**Table 3:** Presence of nanoparticle pellets of three different formulations

Formulation name	Purification condition	Presence of nanoparticle pellet after centrifugation
Formulation-1	Centrifuged at 18 000 rpm for 45 minutes	Absent
Formulation-2	Centrifuged at 18 000 rpm for 45 minutes	Present
Formulation-3	Centrifuged at 18 000 rpm for 45 minutes	Absent

The successful formulation leverages the complementary properties of these lipids: stearic acid, a long-chain saturated fatty acid, provides structural stability and integrity to the SLNs, while lauric acid, a medium-chain fatty acid with lower melting point and higher solubility, enhances biocompatibility and improves drug loading capacity.^25,26^ Together, these naturally occurring lipids create an effective matrix for drug encapsulation while minimizing toxicity risks. This stearic acid–lauric acid combination has previously demonstrated success in controlled delivery systems for other therapeutic agents (doxorubicin and rhodamine B), supporting our selection for STS encapsulation in subsequent experiments.^27^

Following the successful identification of stearic acid and lauric acid as the optimal lipid combination, we proceeded to select an appropriate surfactant for the STS-SLNs formulation. Surfactants are critical for stabilizing nanoparticles by reducing surface tension between the lipid matrix and aqueous phase, preventing aggregation, and facilitating efficient drug encapsulation.^28^ Various surfactants (Table 2) at different concentrations were evaluated using the previously established lipid composition. The formulations were monitored for 24 hours under continuous stirring to assess stability through the absence of phase separation or precipitation (Table 4). While PVA initially demonstrated stability, phase separation became evident after 24 hours at 1% concentration and after 12 hours at 2% concentration. Poloxamer-188 (P-188) and Tween-20 at all tested concentrations (1%, 5%, and 10%) showed immediate phase separation within 3 hours. Similarly, all concentrations of Tween-80 exhibited poor stability.

**Table 4:** Experiment design to identify phase separation or precipitation with different concentrations of surfactant

Name of surfactant	Concentration (%)	Observation time			
		3 hours	6 hours	12 hours	24 hours
PVA	1	−	−	−	+
	2	−	−	+	+
P-188	1	+	+	+	+
Tween-20	1	+	+	+	+
	5	+	+	+	+
	10	+	+	+	+
Tween-80	1	+	+	+	+
	5	+	+	+	+
	10	+	+	+	+
Tween-60	0.5	−	+	+	+
	1	−	+	+	+
	5	−	−	−	−
	10	−	+	+	+

Notably, Tween-60 demonstrated concentration-dependent stability profiles. At 0.5 and 1% concentrations, stability was maintained for 3 hours before phase separation occurred, while at 10% concentration, separation was observed after 6 hours. Significantly, at 5% Tween-60, the stability throughout the entire 24-hour observation period showed no phase separation. Based on these results, we selected 5% Tween-60 as the optimal surfactant for subsequent formulation development. For purification optimization, we evaluated centrifugation at 18 500 rpm for various durations. No nanoparticle pellets were recovered after 30 or 45 minutes of centrifugation. While centrifugation for 1 hour and 30 minutes yielded nanoparticle pellets, the recovery was exceptionally low. To improve the yield, we incorporated an Amicon® Ultra-15 Centrifugal Filter (10 kD, Millipore, MA, USA) into the purification process, performing centrifugation at 4000*g* for 1 hour and 30 minutes. This modified approach successfully yielded nanoparticle pellets while effectively removing excess surfactant and unencapsulated STS in the filtrate. The resulting purified nanoparticles were then subjected to comprehensive physicochemical characterization.

### 3.2. HPLC method development for STS

The analytical techniques based on HPLC were successfully developed for qualitative and quantitative STS determination (Table S2). The HPLC chromatograph showed an intense peak of STS at around 2 minutes recorded at 210 nm. The peak areas of different concentrations of STS solutions were calculated and used to draw a standard calibration curve for each of the 3 runs (Fig. S1). The HPLC method for the qualitative and quantitative analysis of STS was successfully validated according to ICH guidelines (Table S3). The LOD and LOQ values of STS using this method were found to be 0.202283667 ± 0.077047991 and 0.612980667 ± 0.233477517 μg mL^−1^, respectively. The standard equation to determine the concentration of STS in an unknown sample was found to be *y* = 27.354*x* + 3.2019, where *y* is the area of the curve and *x* is the concentration.

### 3.3. Synthesis and physicochemical characterization of STS-SLN formulations

Using the optimized conditions, the STS-SLNs were successfully synthesized using the double emulsion solvent evaporation method. The optimized fabrication protocol involved creating a primary water-in-oil emulsion by incorporating the aqueous STS solution into an organic phase containing stearic acid and lauric acid. This primary emulsion was subsequently dispersed into an external aqueous phase containing 5% Tween-60, resulting in a stable milky colloidal solution. The absence of phase separation following organic solvent evaporation indicated successful formation of stable nanoparticles, which were then purified by centrifugation.

**Fig. 2:**
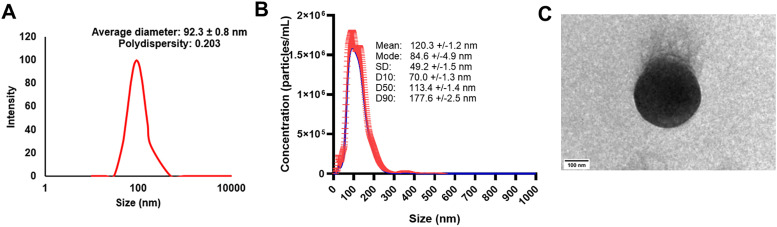
Characterization of STS-SLN formulations. (A) Size distribution of the STS-SLNs analyzed by DLS, (B) size distribution of the STS-SLNs analyzed by nanoparticle tracking analysis, and (C) TEM image of the STS-SLNs exhibiting a spherical shape (scale 100 nm).

Comprehensive physicochemical characterization confirmed the successful development of well-defined nanoparticles. DLS analysis revealed an average particle size of 92.3 ± 0.8 nm with a polydispersity index below 0.3, indicating a homogeneous, monodisperse population (Fig. 2A). More detailed size analysis through NTA produced D10/D50/D90 values of 70.0 ± 1.3/113.4 ± 1.4/177.6 ± 2.5 nm, representing the particle sizes at which 10%, 50%, and 90% of the population fall, respectively (Fig. 2B). TEM confirmed the spherical morphology of nanoparticles with sizes approximating 100 nm, corroborating the DLS and NTA findings (Fig. 2C). Surface charge analysis of STS-SLNs revealed a zeta potential of −13.23 ± 2.07 mV. This negative surface charge originates from the deprotonation of carboxyl (–COOH) groups present in both stearic acid and lauric acid, generating carboxylate ions (–COO^−^) at the nanoparticle surface.^29^ The non-ionic surfactant Tween-60 complements this electrostatic stabilization by providing steric hindrance against particle aggregation. The combination of negative surface charge and steric stabilization creates a stable colloidal system with sufficient interparticle repulsion to prevent aggregation.^30^ This stability is essential for maintaining nanoparticle integrity during storage and administration, ultimately ensuring consistent therapeutic efficacy.^22^

### 3.4. Assessment of encapsulation efficiency (EE) and drug loading (DL) of STS in SLNs

EE and DL represent critical parameters for evaluating the performance of drug delivery systems. High EE indicates successful drug incorporation within the nanoparticle matrix, which enhances stability, reduces premature drug release, and improves bioavailability. Loading capacity reflects the proportion of the drug relative to the total nanoparticle mass, with higher values enabling more efficient delivery of therapeutic doses with minimal carrier material. Characterization of the optimized STS-SLNs revealed an encapsulation efficiency of 45.48 ± 5.87% and a loading capacity of 5.0 ± 0.53%. These values demonstrate the effective incorporation of the hydrophilic STS within the SLN matrix, particularly considering the challenges associated with encapsulating water-soluble compounds in lipid-based carriers. The achieved encapsulation parameters confirm the suitability of the double emulsion solvent evaporation method using the stearic acid–lauric acid combination with Tween-60 as a surfactant for delivering STS to the target site with adequate drug payload.

### 3.5. Study of the release kinetics of STS

**Fig. 3:**
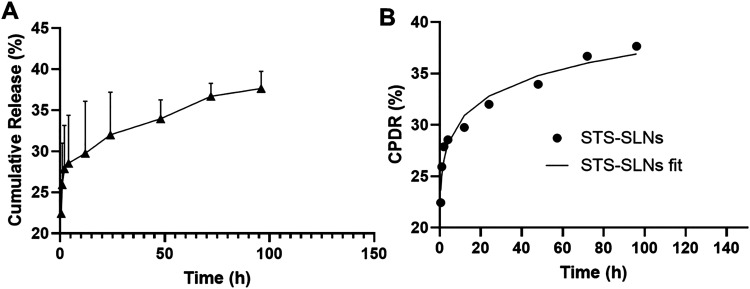
*In vitro* drug release and release kinetics of STS-SLNs. (A) The percent release of STS from SLNs and (B) the fitting of cumulative percent drug release (CPDR) data with the Korsmeyer–Peppas model. Here, STS-SLNs legend show STS released from the solid lipid nanoparticles.

Release kinetics represent a fundamental parameter for evaluating drug delivery systems, with pharmacopeial guidelines emphasizing the importance of controlled release studies that account for receiving medium characteristics, agitation conditions, sampling intervals, and analytical methodology.^31^ To determine the release behavior of STS from our optimized SLNs, we conducted *in vitro* release studies in phosphate-buffered saline (pH 7.4) over four days. The release profile demonstrated a sustained pattern with a maximum cumulative release of 37.0 ± 0.55% of the encapsulated STS after 96 hours (Fig. 3A). This controlled release behavior is particularly advantageous for maintaining therapeutic concentrations at the target site while minimizing systemic exposure and potential side effects. To elucidate the underlying release mechanism, we applied mathematical modeling to experimental data. The release kinetics were best described by the Korsmeyer–Peppas model, with an excellent correlation coefficient (*R*^2^ = 0.9951) (Fig. 3B). This model provides insights into the drug release mechanism through the release exponent (*n*). The calculated *n* value of 0.09 for STS-SLNs indicates that the release follows Fickian diffusion, where drug release is primarily governed by concentration gradient-driven diffusion through the lipid matrix. The release constant (*K*_1_) of 25.04 further characterizes the rate and extent of drug release from the formulation (Table S4). This controlled-release profile, maintained over several days, offers significant advantages for potential inner ear drug delivery applications, where sustained therapeutic concentrations are essential for protecting cochlear hair cells against cisplatin-induced ototoxicity.^20^

### 3.6. Stability and freeze-drying studies

**Fig. 4:**
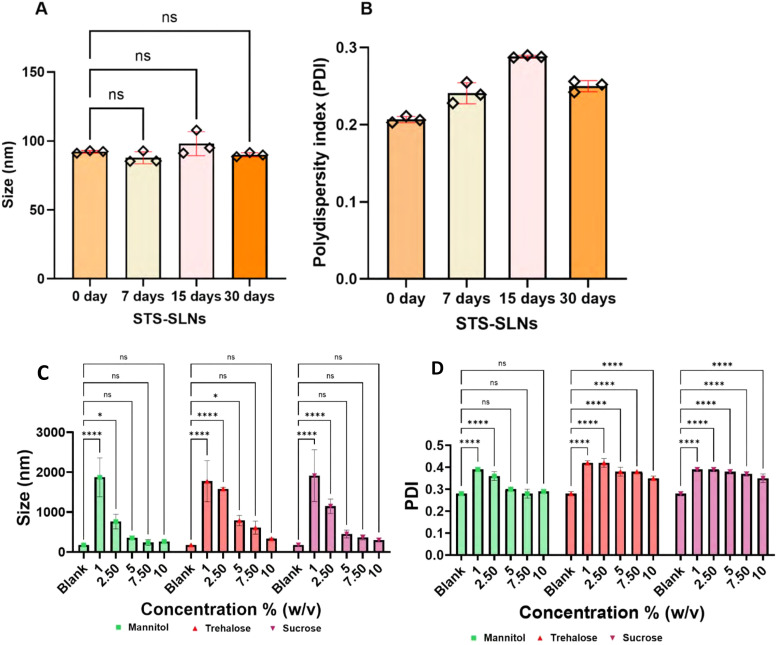
Storage stability of STS-SLNs: size and polydispersity index (PDI) were used as markers to assess the structural integrity of STS-SLNs during storage at 4 °C. The study was conducted at various time intervals following preparation, including 0, 7, 15, and 30 days. Regarding the size (A) and PDI (B) values of the NP preparations, there was no noticeable variation. One-way ANOVA was used to analyze the data for each group (*n* = 3). The effect of different cryoprotectants (mannitol, trehalose, and sucrose) at varying concentrations (1, 2.5, 5, 7.5, and 10% w/v) on particle size (C) and PDI (D) of lyophilized STS-SLNs after reconstitution was studied. Mannitol at 10% preserved nanoparticle stability most effectively, while sucrose and trehalose showed greater variability. The data were analyzed using two-way ANOVA and the group sample means were compared with the blank sample (non-lyophilized) by Dunnett's multiple comparison *post hoc* test. A family-wise alpha of 0.05 (95% CI) was applied, and all comparisons showed no significant differences (*p* > 0.05), indicating that each cryoprotectant maintained NP size and PDI within an optimal range.

The long-term stability of pharmaceutical formulations is essential for ensuring consistent therapeutic efficacy throughout their life. To evaluate the physical stability of the optimized STS-SLNs, we conducted a comprehensive stability study over a one-month period under refrigerated storage conditions (4 °C) in deionized water. Periodic assessment of critical physical parameters using DLS revealed the remarkable stability of the nanoformulation. Particle size measurements showed negligible variations throughout the 30-day study period, maintaining the initial nanometer dimensions (Fig. 4A). Similarly, the polydispersity index remained consistently below 0.3, indicating preservation of the homogeneous size distribution and absence of aggregation (Fig. 4B). The exceptional stability of the STS-SLNs can be attributed to the complementary properties of the lipid components. Stearic acid, a long-chain saturated fatty acid, provides a rigid crystalline structure that serves as a protective matrix for the encapsulated drug, preventing degradation and leakage. This structural integrity is balanced by lauric acid, a medium-chain fatty acid that imparts appropriate flexibility and fluidity to the lipid matrix, enhancing drug accommodation and distribution within the nanoparticle. The synergistic effect of these lipids creates a stable core resistant to aggregation and recrystallization processes that typically compromise nanoparticle stability.^32^ Furthermore, the presence of Tween-60, which reduces surface tension and creates a steric barrier against particle agglomeration, contributes to formulation stability. These results demonstrate that the developed STS-SLNs maintain their physicochemical properties during refrigerated storage for at least one month, supporting their potential application as a pharmaceutical formulation with adequate shelf stability.^33^

To evaluate the feasibility of lyophilization, STS-SLNs were lyophilized in the presence of different cryoprotectants (mannitol, trehalose, and sucrose) at various concentrations (1, 2.5, 5, 7.5, and 10% w/v), and the formulations were analyzed for particle size and PDI following reconstitution (Fig. 4C and D). Particle size analysis revealed that mannitol provided the most effective stabilization, maintaining nanoparticles below 250 nm with low variability. In contrast, trehalose afforded moderate protection, while sucrose was the least effective, with a significant size increase at higher concentrations. PDI values supported these findings, showing much lower variability and more uniform particle distribution compared to trehalose- and sucrose-treated formulations.

These findings indicate the importance of cryoprotectant selection to preserve the colloidal stability of SLNs throughout the lyophilization process. While trehalose is widely reported as an effective stabilizer for nanoparticles, our study indicates that mannitol works better than trehalose and sucrose. Importantly, the ability of mannitol (10%) to preserve the particle size and PDI^34^ suggests its potential for enabling long-term storage and clinical translation of STS-SLNs as a stable lyophilized formulation.

### 3.7. Cellular internalization of STS-SLNs

**Fig. 5:**
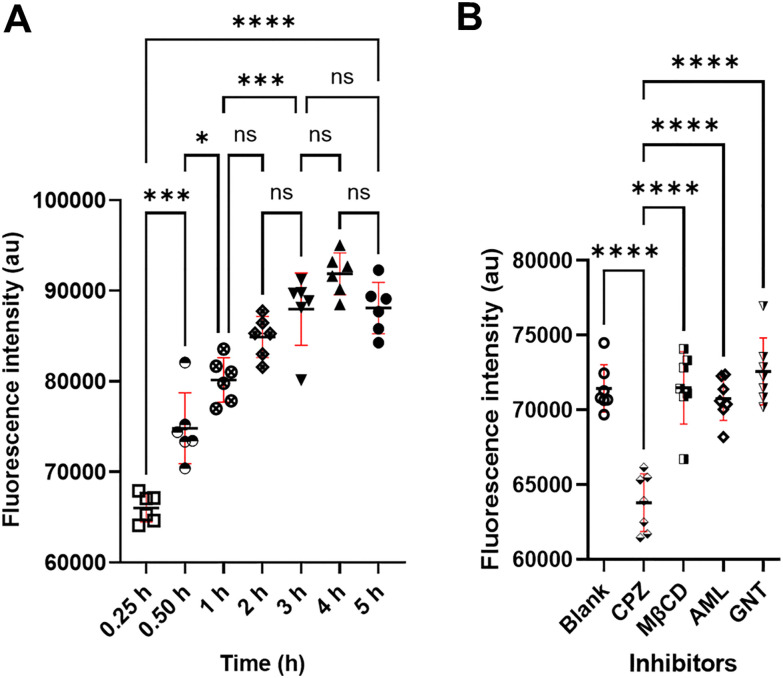
Cellular intracellular study of STS-SLNs. (A) Time required for cellular internalization: the graph displays the fluorescence intensities of the extracted media following the lysis of the cells that were incubated with C-6-NPs for varying times (0.25, 0.50, 1, 2, 3, 4, and 5 h). After 1 hour of incubation, the cells displayed considerable cellular internalization. The highest level of internalization was noted within 1 hour of incubation. Over 90% cellular uptake within 1 hour is indicated by the lack of a discernible difference in the fluorescence intensity between 1 and 5 hours. For STS-SLNs to internalize into cells, the ideal incubation period is therefore between 0.50 and 1 hour. (Using Sidak's multiple comparison *post hoc* test, the groups were compared using a one-way ANOVA. **p ≤ 0.005; *p ≤ 0.05; ‘*n* = 6’, asterisks: ****p < 0.0001); ns = not significant.) (B) The understanding of the internalization pathway within cells. The graph illustrates internalization of C-6-NPs following treatment with particular inhibitors of the internalization pathway. The clathrin-dependent endocytosis inhibitor chlorpromazine (CPZ) significantly inhibits endocytosis in this situation. On the other hand, amiloride (AML; macropinocytosis inhibitor), genistein (GNT; caveolin mediated endo-cytosis inhibitor), and methyl-β-cyclodextrin (MβCD; cholesterol-dependent lipid rafting inhibitor) present differences (Sidak's multiple comparison posthoc test was used to compare the groups using a one-way ANOVA. ‘*n* = 8’ asterisks: ns = not significant; ***p < 0.001).

Cellular uptake plays a crucial role in determining drug efficacy, as therapeutic agents must enter cells to exert their intended pharmacological effects. To investigate this critical process for SLNs, we employed fluorescence microscopy to visualize the internalization of SLNs labeled with the fluorescent marker Coumarin 6 (C-6) in HEI-OC1 cells. Our time-course analysis over a 5-hour period revealed that C-6-NP internalization follows a time-dependent profile. Fluorescence intensity measurements demonstrated progressive uptake with maximum cellular accumulation occurring at the 1 h timepoint (Fig. 5A). This temporal pattern suggests an optimal incubation period for ensuring maximal delivery of STS-SLNs to auditory cells, which could be valuable for establishing effective dosing regimens in future therapeutic applications.

To elucidate the specific endocytic pathway responsible for SLN internalization, we conducted mechanistic studies using selective pathway inhibitors. Cells pretreated with chlorpromazine (CPZ), a well-established inhibitor of clathrin-dependent endocytosis, displayed significantly reduced fluorescence intensity compared to untreated control cells. This marked reduction in uptake indicates that clathrin-mediated endocytosis serves as the predominant route for SLN entry into HEI-OC1 cells. In contrast, when cells were pretreated with inhibitors targeting alternative endocytic pathways—specifically amiloride (AML) for macropinocytosis, genistein (GNT) for caveolin-mediated endocytosis, and methyl-β-cyclodextrin (MβCD) for cholesterol-dependent lipid raft-mediated endocytosis—no substantial reduction in fluorescence intensity was observed (Fig. 5B). These findings collectively provide compelling evidence that our SLNs are selectively internalized *via* clathrin-dependent endocytosis. These findings provide critical insights into the cellular processing of our STS-SLNs and may guide future formulation refinements to enhance drug delivery to auditory cells.

### 3.8. Cytoprotective effects of the STS-SLNs against cisplatin-induced ototoxicity

#### 3.8.1. MTT cell viability assessment

**Fig. 6:**
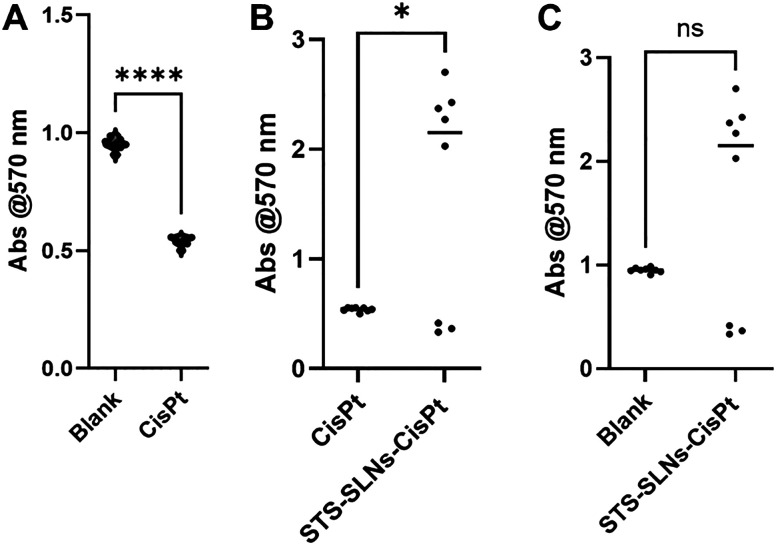
Cytoprotective effect of STS-SLNs against cytotoxicity induced by CisPt. (A) Significant reduction in absorbance (cell viability) in cells treated with CisPt compared to the blank control, (B) significant increase in absorbance in CisPt-treated cells co-treated with STS-SLNs compared to CisPt-only treated cells, indicating cytoprotection. (C) There was no significant difference (ns) in absorbance between the blank and STS-SLNs groups, indicating no cytotoxicity of STS-SLNs on their own. Analysis was done with a *T*-test. Absorbance measured at 570 nm. (****p < 0.0001, *p < 0.05.)

To assess the cytoprotective effect of the STS-SLNs against CisPt-induced cytotoxicity, a cell viability assay was performed by measuring the absorbance at 570 nm. As shown in Fig. 6A, CisPt treatment significantly reduced absorbance levels compared to the blank control group indicating severe cytotoxicity. However, cells treated with STS-SLNs exhibited a significant increase in absorbance compared to the CisPt-only group suggesting a partial restoration of cell viability (Fig. 6B). Furthermore, there is no significant difference in absorbance between the STS-SLN treated group and the blank control, indicating that STS-SLNs alone did not induce cytotoxicity (Fig. 6C).

These findings highlight the potential of the STS-SLNs as an otoprotective intervention during CisPt chemotherapy effectively mitigating drug-induced damage while maintaining their own biocompatibility.

#### 3.8.2. Live–dead cell analysis

To further corroborate the cytoprotective effects observed in the MTT assay, a fluorescence-based live–dead cell analysis was performed on HEI-OC1 cells subjected to various treatment regimens. This dual-staining approach provides direct visualization of cellular viability by differentiating between live and dead cells, offering crucial insights into cell health under different experimental conditions.^35^

**Fig. 7:**
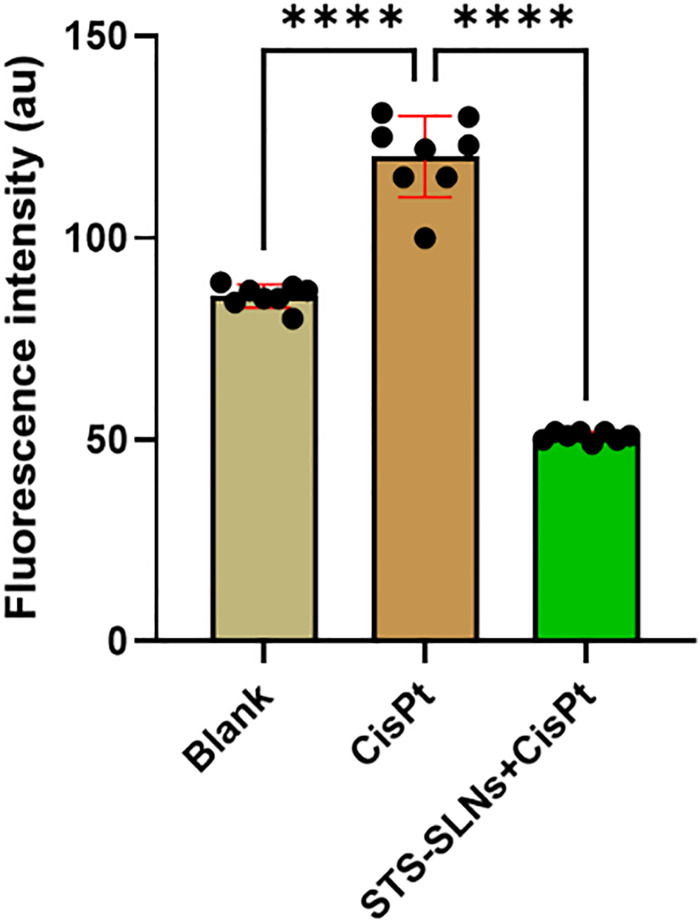
Live–dead cell analysis showing fluorescence intensity of cells treated with blank, CisPt, and STS-SLNs. Here, CisPt significantly increased cell death, while STS-SLNs reduced cytotoxicity. One-way ANOVA was used to analyze the data for each group (*n* = 8) (****p < 0.0001).

Consistent with the MTT results, CisPt treatment induced significant cell death, evidenced by elevated fluorescence intensity compared to untreated controls. Importantly, administration of the STS-SLNs markedly reduced this fluorescence signal, indicating substantial protection against CisPt-induced cytotoxicity (Fig. 7). The live–dead cell imaging provided visual confirmation of the enhanced cell survival in the STS-SLN treatment groups, reinforcing the potential of this nanoformulation as an effective strategy for preserving auditory cell viability during CisPt therapy.

#### 3.8.3. Intracellular ROS scavenging capability study

**Fig. 8:**
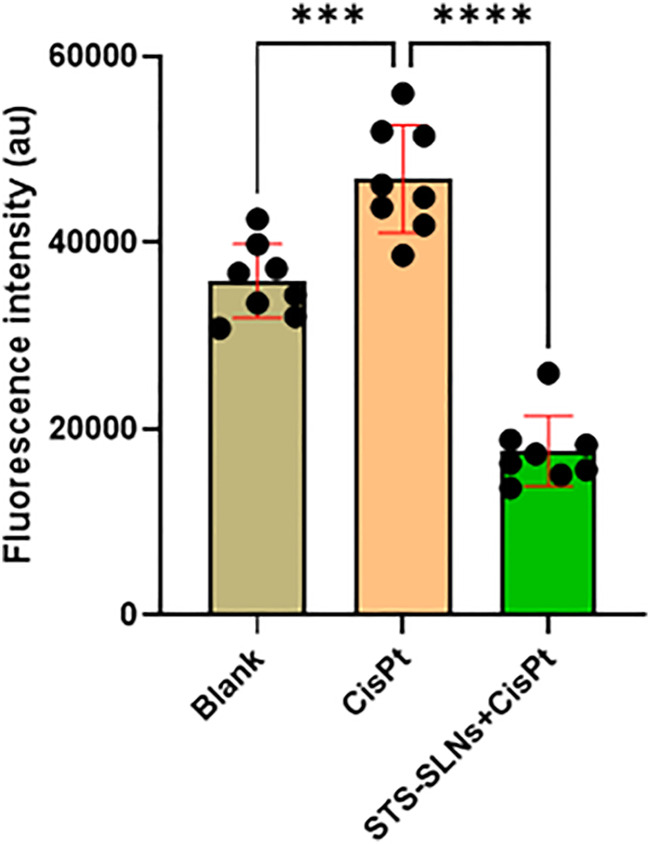
DCFH_2_-DA analysis showing the fluorescence intensity of ROS in cells treated with blank, CisPt, and STS-SLNs. CisPt significantly increased ROS production, while STS-SLN treatment significantly reduced ROS levels. One-way ANOVA was used to analyze the data for each group (*n* = 8), (***p < 0.001, ****p < 0.0001).

To evaluate the potential of the STS-SLNs in mitigating CisPt-induced oxidative stress, we employed the widely recognized DCFH_2_-DA assay method for both qualitative and quantitative assessment of intracellular reactive oxygen species (ROS).^20^ Following sequential treatment with cisplatin (CisPt) and STS-SLNs, cells were incubated with DCFH2-DA for 30 minutes to allow for ROS detection. Our analysis revealed significantly elevated fluorescence intensity of the CisPt treated cells compared to the control cell group, confirming the pro-oxidant effects of CisPt therapy. This significant increase in fluorescence directly correlates with enhanced intracellular ROS generation, which is a primary mechanism underlying MIO. Notably, cells treated with STS-SLNs following CisPt exposure exhibited significantly decreased fluorescence intensity compared with the CisPt only group. This significant decrease in fluorescence co-relatable with detectable ROS levels provides compelling evidence for the robust antioxidant capabilities of our STS-SLN formulation (Fig. 8). The observed ROS-scavenging effect suggests that STS effectively neutralizes CisPt-generated free radicals when delivered *via* SLNs. These results indicate that STS-SLNs are a promising therapeutic approach for preventing CisPt-induced ototoxicity, as they not only enhance intracellular defense mechanisms but also provide efficient drug delivery.

#### 3.8.4. Assessment of molecular mechanisms using western blot analysis

**Fig. 9:**
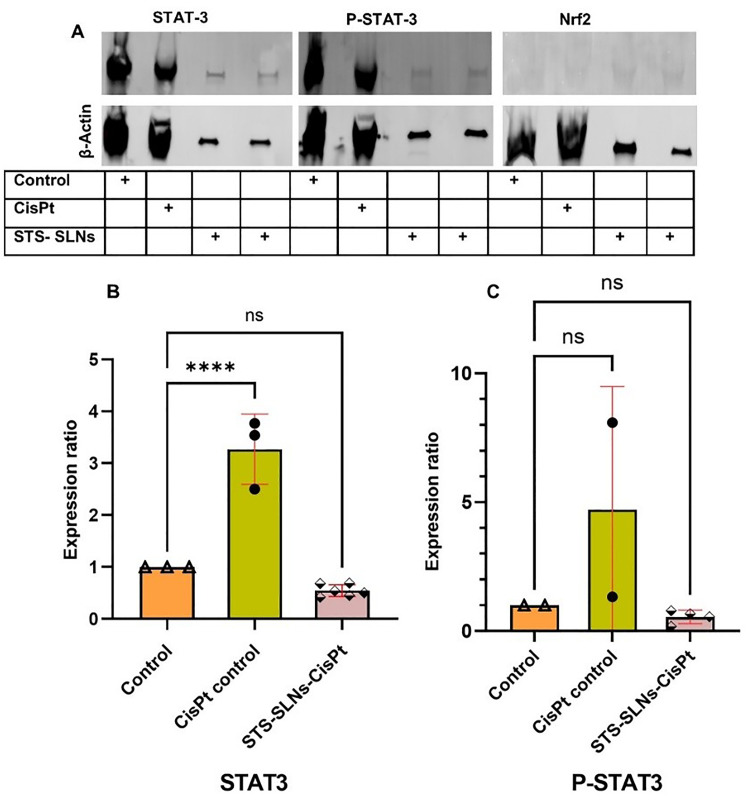
Western blot analysis of STAT3, P-STAT3, and Nrf2 expression in HEI-OC1 cells treated with STS-SLNs in the presence of CisPt. (A) Western blot images showing the expression levels of STAT3, P-STAT3, and Nrf2, with β-actin as the loading control. No detectable Nrf2 was observed across all conditions. (B) Quantifications of STAT3 and (C) P-STAT3 expression ratios normalized to β-Actin. STAT3 expression was significantly upregulated in the CisPt control group, while STS-SLN treatment reduced STAt3 expression. P-STAT3 levels showed variability across conditions. Data are represented as mean ± Standard Error of the Mean (SEM). Statistical analysis was performed using one-way ANOVA; *p < 0.001, ns = not significant.

Western blot analysis was performed to elucidate the molecular mechanisms underlying the protective effects of STS-SLNs against cisplatin-induced ototoxicity in HEI-OC1 cells. We evaluated the expression levels of STAT3 and P-STAT3, key mediators in inflammatory and cell survival pathways. Protein expression was quantified using densitometry, normalized to β-actin as a loading control, and expressed as the ratio of target protein to loading control (Fig. 9). STAT3 expression was significantly upregulated in the CisPt control group (ratio: 1.38–2.11) compared to the untreated control (0.30–0.56) (Table S5). This substantial increase suggests activation of STAT3-mediated inflammatory signaling in response to CisPt exposure, consistent with previous reports implicating inflammation in CisPt-induced toxicity.^36^ Notably, treatment with STS-SLNs led to a marked reduction in STAT3 expression (ratio range: 0.20–0.25), indicating that the STS-SLN nanoformulation effectively suppresses STAT3 activation (Fig. 9B). Adjusted STAT3 ratios further confirmed this trend, with a 3.54–3.77-fold increase in CisPt-treated cells, whereas STS-SLN treatment significantly reduced STAT3 expression (adjusted ratio: 0.42–0.54). This demonstrates the potent anti-inflammatory potential of the STS-SLN nanoformulation.

Similarly, P-STAT3 levels showed a similar trend but with more pronounced effects (Table S6). CisPt treatment dramatically increased P-STAT3 expression (ratio: 2.67) compared to the control (ratio range: 0.176–0.33), representing an 8.1-fold increase when adjusted to the control values. STS-SLN administration substantially reduced P-STAT3 levels (ratio range: 0.06–0.22; adjusted ratio range: 0.2–0.7), indicating effective inhibition of STAT3 phosphorylation and subsequent activation (Fig. 9C). Furthermore, the ratio of P-STAT3 to STAT3 was calculated in the control and treatment groups. The ratio of P-STAT3/STAT3 in the control group was found to be 1, which was increased to 1.4 in the CisPt treated group confirming the overexpression of P-STAT3. In the treatment group (CisPt + STS-SLNs), the ratio was found to be 1 which suggested the normalization of P-STAT3 expression after treatment of the cells with STS-SLNs (Table S7). These results confirmed that STS-SLN treatment has an effect on the STAT signaling pathway. Despite examining Nrf2 expression (Fig. 9A), a key transcription factor in antioxidant defense pathways, no detectable Nrf2 band was observed across any experimental conditions, suggesting that the protective effects of STS-SLNs may be independent of Nrf2-mediated antioxidant responses in this cellular model. These findings provide compelling molecular evidence that STS-SLNs exert their otoprotective effects at least partially through the suppression of STAT3 signaling pathways. By inhibiting both total STAT3 expression and its activating phosphorylation, STS-SLNs may effectively mitigate the inflammatory cascade triggered by CisPt exposure in auditory cells.

HEI-OC1 cells are a well-established *in vitro* model of auditory sensory cells, commonly used in proof-of-concept studies to evaluate new drug candidates and delivery formulations aimed at mitigating inner ear hearing loss.^37^ Thus, our preliminary study has been focused at the cellular and cell signaling levels using these cells. The next phases of preclinical investigation will incorporate *ex vivo* cochlear explant systems and *in vivo* animal models to assess nanocarrier transport across the round window membrane, perilymph distribution,^38^ and interactions within the cochlear microenvironment.^39^

## 4. Conclusions

The development, optimization, and characterization of STS-SLNs presented in this study demonstrate a promising approach for mitigating MIO. The use of a double-emulsion solvent evaporation technique enabled the adequate encapsulation of STS within SLNs, resulting in a stable formulation with desirable physicochemical properties, including optimal particle size, polydispersity index, and zeta potential. The combination of stearic acid and lauric acid as the lipid matrix, along with the appropriate selection of surfactants, contributed to the stability and controlled release profile of the optimized formulation. The optimized cryoprotectant condition (10% mannitol) preserved particle size and polydispersity after reconstitution, indicating good lyophilization tolerance of the STS-SLN formulation. The *in vitro* release studies confirmed a sustained release of STS, which is critical for maintaining therapeutic efficacy over extended periods. Furthermore, the cellular uptake experiments demonstrated that the STS-SLNs were effectively internalized by HEI-OC1 cells *via* a clathrin-dependent endocytic pathway, indicating potential for inner ear drug delivery. It demonstrated efficient cytoprotection in HEI-OC1 cells against toxicity caused by CisPt. Furthermore, intracellular ROS levels were decreased by the STS-SLN formulation, highlighting its potential to lessen oxidative stress brought on by CisPt. The formulation's stability over time at low temperatures further supports its viability for clinical applications. Western blot analysis confirmed the inhibitory effects of STS-SLNs on STAT3 expression, highlighting its potential role in suppressing inflammatory pathways associated with MIO. Overall, this work concludes with the comprehensive development and evaluation of the STS-SLN delivery system against medication-induced hearing loss using CisPt as a model drug.

## Consent for publication

All authors agreed to publish this manuscript.

## Author contributions

B. C. data curation, formal analysis, investigation, methodology, writing – original draft, review and editing, and validation. N. S. T. conceptualization, formal analysis, methodology, writing – review and editing, and validation. A. D. J. formal analysis, resources, writing – review and editing. V. A. conceptualization, formal analysis, funding acquisition, project administration, resources, supervision, writing – review and editing.

## Conflicts of interest

The authors declare no competing interests.

## Abbreviations

AML: Amiloride
CPZ: Chlorpromazine
DLS: Dynamic light scattering
EE: Encapsulation efficiency
GNT: Genistein
HEI-OC1: House Ear Institute-Organ of Corti 1 cells
HL: Hearing loss
HPLC: High-performance liquid chromatography
DL: Drug loading
MβCD: Methyl-β-cyclo-dextrin
MIO: Medication-induced ototoxicity
mPEG: methoxy poly(ethylene glycol)
Nrf2: Nuclear factor erythroid 2-related factor 2
PCL: Polycaprolactone
PDI: Polydispersity
PI: Propidium iodide
PLA: Polylactic acid
P-STAT3: Phosphorylated form of STAT3
PVA: Polyvinyl alcohol
STAT3: Signal transducer and activator of transcription 3
STS: Sodium thiosulphate
STS-SLNs: STS-loaded solid lipid nanoparticles
TEA: Triethylamine
TEM: Transmission electron microscopy

## Supplementary Material

TB-013-D5TB01324K-s001

## Data Availability

All data generated or analyzed during this study are included in this published article in the main text or supplementary information (SI). Any additional information is available from the corresponding author on reasonable request. Supplementary information contains all supporting data regarding formulation optimization, HPLC method development of STS, release kinetics of STS, WB analysis, expression levels of STAT3 and P-STAT3. See DOI: https://doi.org/10.1039/d5tb01324k.
