# Supplementary material for: Rationally designed sodium thiosulfate-loaded solid lipid nanoparticles for inner ear delivery and prevention of medication-induced ototoxicity
Source: J Mater Chem B. 2025 Oct 27;13(45):14690–703. doi: 10.1039/d5tb01324k (PMC12557298; doi:10.1039/d5tb01324k)
Supplement: TB-013-D5TB01324K-s001 [file TB-013-D5TB01324K-s001.pdf]

Supplementary Information (SI) for Journal of Materials Chemistry B.
This journal is © The Royal Society of Chemistry 2025

Rationally Designed Sodium Thiosulfate-Loaded Solid Lipid Nanoparticles for Inner Ear Delivery and Prevention of Medication-induced Ototoxicity

Brototi Chakrabarty[1], Neeraj S. Thakur[1], Aditya D. Joshi[2], Vibhuti Agrahari[1]*

[1]Department of Pharmaceutical Sciences, The University of Oklahoma, 1110 North Stonewall Avenue, Oklahoma City, OK, 73117, USA

[2]Department of Veterinary Physiology and Pharmacology, Texas A& M University, College Station, TX, 77843, USA

*Corresponding Author:

vibhuti-agrahari@ouhsc.edu

ORCID: Neeraj S. Thakur: 0000-0003-1758-0737

ORCID: Aditya D. Joshi: 0000-0002-5208-5144

ORCID: Vibhuti Agrahari: 0000-0003-1884-1644

Contents

S1. Formula optimization 3

S2. HPLC method development STS…………………………………………………..................3

S3. Study of the Release Kinetics of STS...5

S4. Western blot analysis…….…………………………………………………………………4

S5: Expression level of STAT3……………………………………………………………….5

S6: Expression level of P-STAT3……………………………………………………………6

S7: Expression ratio of P-STAT3 and STAT3………………………………………………..6

S1. Formulation optimization

Table S1: Composition of optimized formulation

Composition	Amount
Stearic acid	10 mg
Laric acid	10 mg
Tween- 60	5 %
STS	2 mg
Water	4 mL

S2. HPLC method development of STS

Table S2. HPLC method parameters for STS qualitative and quantitative analysis

Method parameters	STS
Mobile Phase (MP)	A: Methanol (100%)<br><br>B: 0.1%TEA pH 7.4<br><br>Ratio of A: B = 15:85
Column	C-18 Phenomenex Columbus 5µ, 100×4.8 mm, 25 °C
Injection volume	10 µL
Flow Rate	0.5 mL/min
Wavelength	210 nm
Acquisition time	4 min

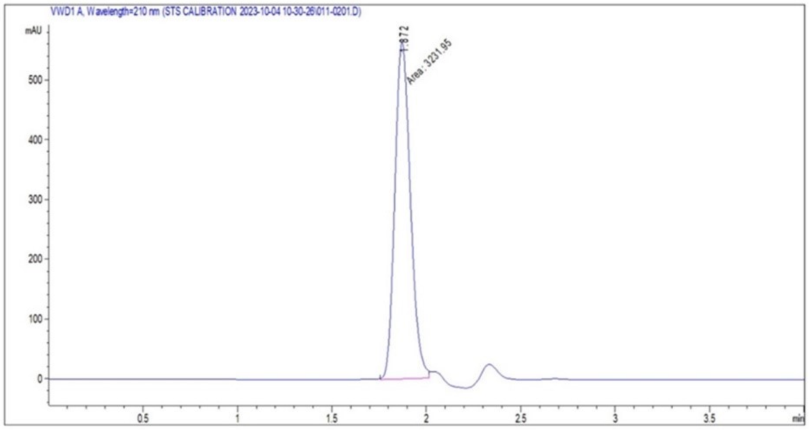

Figure S1. HPLC chromatogram of STS

Table S3. HPLC validation table for the determination of LOD and LOQ of STS.

	**Symbol**	**STS1**	**STS2**	**STS3**	**Avg**	**SD**
Slope	S	23.58732	23.95858	34.57222	27.354	6.237724
Intercept	b	4.36111	4.12222	5.57222	3.2019	0.777427
Number of tests	N	3	3	3		
SE of Intercept		1.1508	0.608393	1.71076	1.4230109	0.551207
SE of Regression	σ	1.6999	0.898656	2.52696	3.7665069	0.814186
LOD (µg/mL)	$LOD = \frac{3.3\sigma}{S}$	0.23782	0.123779	0.245252	**0.202284**	0.068088514
LOQ (µg/mL)	$LOD = \frac{10\sigma}{S}$	0.720666	0.375089	0.743187	**0.612980**	0.206327731
LOD: Limit of Detection; LOQ: Limit of Quantitation; SE: Standard Error						

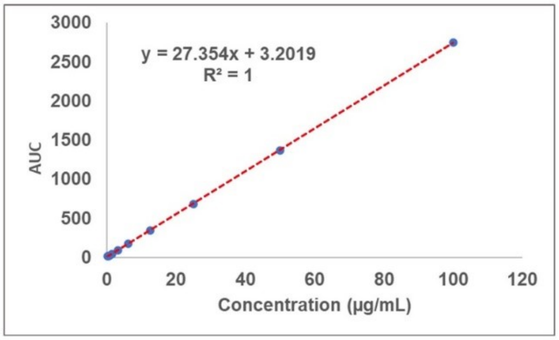

Figure S2: Standard curve for STS quantification

S3: Study of the Release Kinetics of STS

Table S4: Values of the Korsmeyer-Peppas model fit for the STS release from optimized SLNs

Model parameters	STS nanoformulation
K_1	25.04
n	0.09
R^2	0.9951

S4: Western blot analysis

Recipe for 2X blue dye (10 mL)

1. 0.5 M Tris HCl pH 6.8 (2.5 mL)
2. 10% SDS (4 mL)
3. Glycerol (2 mL)
4. 0.1% Bromophenol Blue (0.3 mL)
5. 2- mercaptoethanol (1 mL)
6. Deionized water (0.2 mL)

STAT3	Control			Cisplatin control			STS-SLNs					
**Target protein**	241126810	198259908	199027140	367141732	366043780	210231254	56440062	41722690	14528004	59175744	43127300	11999676
**Loading control**	617281544	351202224	592,669,060	265678608	173537595	242,892,804	287900996	177393480	62581234	278434136	169319280	51089100
**Ratio**	0.39	0.56	0.34	1.38	2.11	0.87	0.20	0.24	0.23	0.21	0.25	0.23
**Adjusted ratio**	1.00	1.00	1.0	3.54	3.77	2.5	0.50	0.42	0.7	0.54	0.45	0.7

S5: Expression level of STAT3

S6: Expression level of P-STAT3

P-STAT3	Control		Cisplatin control		STS-SLNs			
**Target protein**	97523730	137,650,156	112765437	159,609,420	14073345	15,733,575	17536432	8,824,680
**Loading control**	554542800	420,429,702	481,973,135	59,787,130	145936505	70,734,720	126116208	149,281,634
**Ratio**	0.176	0.33	0.234	2.67	0.096	0.22	0.139	0.06
**Adjusted ratio**	1.00	1.0	1.33	8.1	0.55	0.7	0.79	0.2

S7: Expression ratio of P-STAT3 and STAT3

Groups	P-STAT3/STAT3

Control	1.00
CisPt	1.44
STS-SLNs-CisPt	1.00
